# Characteristics and practices of school-based cluster randomised controlled trials for improving health outcomes in pupils in the United Kingdom: a methodological systematic review

**DOI:** 10.1186/s12874-021-01348-0

**Published:** 2021-07-26

**Authors:** Kitty Parker, Michael Nunns, ZhiMin Xiao, Tamsin Ford, Obioha C. Ukoumunne

**Affiliations:** 1 NIHR Applied Research Collaboration South West Peninsula (PenARC), University of Exeter, Room 2.16, South Cloisters, St Luke’s Campus, 79 Heavitree Rd, Exeter, EX1 2LU UK; 2 College of Medicine and Health, University of Exeter, St Luke’s Campus, Heavitree Road, Exeter, EX1 2LU UK; 3 School of Health and Social Care, University of Essex, Colchester, CO4 3SQ UK; 4 Department of Psychiatry, University of Cambridge, L5 Clifford Allbutt Building, Cambridge Biomedical Campus Box 58, Cambridge, CB2 0AH UK

**Keywords:** Child and adolescent health, Cluster randomised trials, Public health, Randomised trials, Research methods, Schools, Systematic review

## Abstract

**Background:**

Cluster randomised trials (CRTs) are increasingly used to evaluate non-pharmacological interventions for improving child health. Although methodological challenges of CRTs are well documented, the characteristics of school-based CRTs with pupil health outcomes have not been systematically described. Our objective was to describe methodological characteristics of these studies in the United Kingdom (UK).

**Methods:**

MEDLINE was systematically searched from inception to 30^th^ June 2020. Included studies used the CRT design in schools and measured primary outcomes on pupils. Study characteristics were described using descriptive statistics.

**Results:**

Of 3138 articles identified, 64 were included. CRTs with pupil health outcomes have been increasingly used in the UK school setting since the earliest included paper was published in 1993; 37 (58%) studies were published after 2010. Of the 44 studies that reported information, 93% included state-funded schools. Thirty six (56%) were exclusively in primary schools and 24 (38%) exclusively in secondary schools. Schools were randomised in 56 studies, classrooms in 6 studies, and year groups in 2 studies. Eighty percent of studies used restricted randomisation to balance cluster-level characteristics between trial arms, but few provided justification for their choice of balancing factors. Interventions covered 11 different health areas; 53 (83%) included components that were necessarily administered to entire clusters. The median (interquartile range) number of clusters and pupils recruited was 31.5 (21 to 50) and 1308 (604 to 3201), respectively. In half the studies, at least one cluster dropped out. Only 26 (41%) studies reported the intra-cluster correlation coefficient (ICC) of the primary outcome from the analysis; this was often markedly different to the assumed ICC in the sample size calculation. The median (range) ICC for school clusters was 0.028 (0.0005 to 0.21).

**Conclusions:**

The increasing pool of school-based CRTs examining pupil health outcomes provides methodological knowledge and highlights design challenges. Data from these studies should be used to identify the best school-level characteristics for balancing the randomisation. Better information on the ICC of pupil health outcomes is required to aid the planning of future CRTs. Improved reporting of the recruitment process will help to identify barriers to obtaining representative samples of schools.

## Background

Cluster randomised trials (CRTs) are studies in which groups, or clusters, of individuals are allocated to trial arms rather than the individuals themselves [1]. The clusters may be geographic areas, health organisations or social units. CRTs are used when the intervention is delivered to the entire cluster or there is a chance of contamination between trial arms if individuals are randomised [2].

CRTs can be more complex to design and analyse than individually randomised controlled trials. The most documented methodological consideration for CRTs is that observations on participants from the same cluster are more likely to be similar to each other than those on participants from different clusters [2]. This similarity is quantified by the intra-cluster correlation coefficient (ICC), defined as the proportion of the total variability in the trial outcome that is between clusters as opposed to between individuals within clusters [3]. The statistical dependence between observations within clusters needs to be taken account of when calculating the sample size and analysing data in CRTs [1]. The use of standard methods may result in the sample size being too small to detect the intervention effect, and analysis results that exaggerate the evidence for a true intervention effect. Estimates of the ICC or coefficient of variation of clusters for the outcome from previous studies are required to calculate the design effect, the factor by which the number of individuals that would be required in an individually randomised trial needs to be inflated to account for within-cluster correlation in the sample size calculation. In addition, when calculating the sample size in CRTs, a degrees of freedom correction should be incorporated to take account of the uncertainty with which variability in the outcome across clusters is estimated in the analysis [4], and a further inflation of the sample size should be considered to allow for loss of efficiency that results from recruiting unequal numbers of participants from the clusters[5]. When estimating the intervention effect from the resulting trial data the main analytical approaches are to either apply standard statistical methods to summary statistics that represent the cluster response (cluster-level analyses) or use methods at the individual participant level that account for within-cluster correlation in the model or by weighting the analysis. Another important methodological consideration in CRTs is the potential for recruitment bias that might occur in studies where the participating individuals are recruited after the clusters are randomised. Finally, when using meta-analysis to pool findings from studies that use the CRT design, there is the need to consider how best to incorporate estimated effects from studies that did not allow for clustering in the analysis, and consider the extent to which differences in the types of clusters that were randomised are a source of heterogeneity. These considerations are detailed in several textbooks [1, 2, 6–8].

CRTs are increasingly used to evaluate non-pharmacological interventions for improving child health outcomes [9–11]. Although the use of CRTs to evaluate the effectiveness of interventions for improving educational outcomes is long established [12, 13], their use to evaluate health interventions in schools is more recent [10]. Schools provide a natural environment to recruit, deliver public health interventions to and measure outcomes on children, due to the amount of time they spend there [10]. Cluster randomisation is consistent with the natural clustering found within school settings (i.e., classrooms within year groups within schools). School-based CRTs share common challenges with other settings, but specific considerations may be more challenging when schools are randomised, for example, consent procedures [10, 14].

In 2011, a methodological systematic review on the characteristics and quality of reporting of CRTs involving children reported a marked increase in such studies [9]; three quarters of the included studies randomised schools. To date, no systematic review has focussed specifically on the characteristics of school-based CRTs for improving pupil health outcomes. Such a review would help identify common methodological challenges, obtain estimates of parameters (e.g., the ICC) that are of use to researchers planning similar trials and inform the design of simulation studies that use synthetic data to evaluate the properties of statistical methods applied in the context of school-based CRTs with health outcomes.

The aim of this methodological systematic review is to describe the characteristics and practices of school-based CRTs for improving health outcomes in pupils in the United Kingdom (UK).

## Methods

This is a systematic review of school-based CRTs with pupil health outcomes that were conducted in the UK. The review was focussed on the UK to align with constraints on available resources and collect richer data on CRT methodology in a single education system.

### Data sources and search methods

The systematic review was registered with PROSPERO (CRD42020201792) and the protocol has been published [15]. After extensive scoping of the subject area, a pragmatic decision was made to search MEDLINE (through Ovid) in order to make the review more time-efficient and align with available resources. MEDLINE was exclusively searched from inception to 30^th^ June 2020 for peer-reviewed articles of school-based CRTs. The search strategy (Table 1) was developed in consultation with information specialists, based on a sensitive MEDLINE search strategy for identifying CRTs [16]. Cluster design-related terms ‘cluster*’, ‘group*’ and ‘communit*’ were combined with the terms ‘random’ and ‘trial’, along with the ‘Schools’ Medical Subject Heading (MeSH) term. The search was limited to English language.

**Table 1:** Systematic review search strategy

**Search strategy**
**Terms for randomised controlled trials:**
1. random:.mp.
2. trial.ab, kw, ti.
**Cluster design-related terms:**
3. “cluster*”.ab, kw, ti.
4. “group*”.ab, kw, ti.
5. “communit*”.ab, kw, ti.
6. 3 OR 4 OR 5
**School MeSH term:**
7. exp Schools/
**Highest precision:**
8. 1 AND 2 AND 6 AND 7
9. 8 limited to English language

### Inclusion and exclusion criteria

The systematic review included school-based definitive CRTs of the effectiveness of an intervention versus a comparison group that evaluated health outcomes on pupils. The population of interest was children in full-time education in the UK. Studies that took place outside the UK were excluded. The pragmatic decision was made to limit the population to educational settings within the UK as it made the review more focussed and applicable to a specific setting. Eligible studies included pupils in pre-school, primary school and secondary school. The types of eligible clusters included schools themselves, year groups, classes, teachers or any other relevant school-related unit. All school types were eligible, including special schools. Any health-related intervention(s) and control groups were considered. The primary outcome had to be related to pupils’ health. Studies for which the primary outcome was not health-based (e.g., academic attainment) were excluded. All types of CRT design were eligible including parallel group, factorial, crossover and stepped wedge studies.

If more than one publication of the primary outcome result for an eligible CRT was identified, a key study (index) report was designated and used for data extraction. Papers that did not report the primary outcome were excluded along with pilot/feasibility studies, protocol/design articles, process evaluations, economic evaluations/cost-effectiveness studies, statistical analysis plans, commentaries and mediation/mechanism analyses.

### Sifting and validation

Two reviewers (KP and OU) independently screened the titles and abstracts of all references (downloaded into Endnote [17]) for eligibility against the inclusion criteria. Any studies for which the reviewers were uncertain of for inclusion were taken to full text screening. Full-text articles were evaluated by the same reviewers based on the inclusion criteria using a pre-piloted coding method. Any discrepancies which could not be resolved through discussion were sent to a third reviewer (ZMX) for a decision.

### Data extraction and analysis

For each eligible study, data were extracted using a pre-piloted form in Microsoft Excel. Data were extracted by two reviewers (KP and OU), and any discrepancies that could not be resolved through discussion were sent to a third reviewer (ZMX) for a final decision. Missing information that was not available in the index papers was sought from corresponding protocol papers and other “sibling” publications.

The items of information extracted are listed as follows:

- *Publication details:* year of publication and journal name.
- *Setting characteristics:* country/region, school level and type of school.
- *Intervention:* health area and intervention type.
- *Primary outcome:* name, health area, reporter of outcome and method of data collection.
- *Study design and analysis methods:* unit of randomisation (i.e., type of cluster), justification for using the cluster trial design, method used to sample schools, method used to balance the randomisation, length and number of follow-ups, design of follow-up (cohort versus repeated cross-sectional design) and method used to account for clustering in the analysis.
- *Sample size calculation:* target sample size (i.e., number of clusters and pupils) and assumptions underlying the sample size calculation (e.g., assumed ICC, percentage loss to follow-up).
- *Ethics and consent procedures:* activities covered by the consent agreements and use of “opt-out” consent.
- *Other study characteristics of methodological interest:* number of clusters and pupils that were recruited and lost to follow-up, estimate of the ICC of the primary outcome.

Study characteristics were described using medians, interquartile ranges (IQRs) and ranges for continuous variables, and numbers and percentages for categorical variables, using Stata software [18]. Formal quality assessment was not performed as it was not an objective of this review to estimate intervention effects in the included studies. Some information relevant to the quality of CRTs was, however, extracted and summarised as part of the review.

## Results

### Search results

**Fig. 1:**
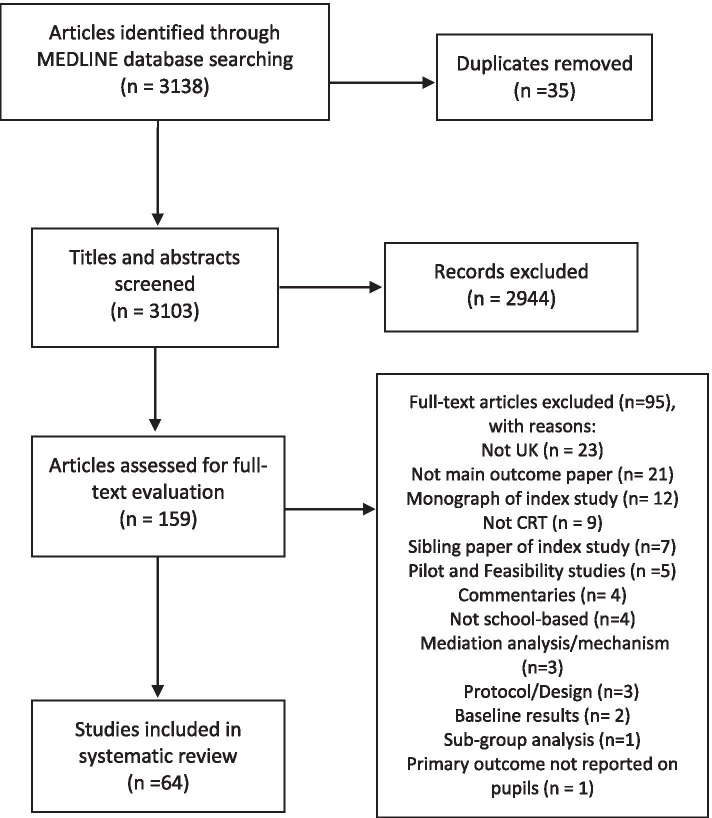
PRISMA flowchart summarising the results of the literature search and screening for eligibility

After deduplication, 3103 articles were identified through MEDLINE, 159 were full-text screened and 64 were included in the review [19–82]. Of 95 excluded studies, 88 did not meet the inclusion criteria, and 7 studies met inclusion criteria but were subsequently excluded because they were sibling reports of an index paper. The PRISMA (Preferred Reporting Items for Systematic Reviews and Meta-Analyses) flow diagram is in Fig. 1.

### Study characteristics

**Fig. 2:**
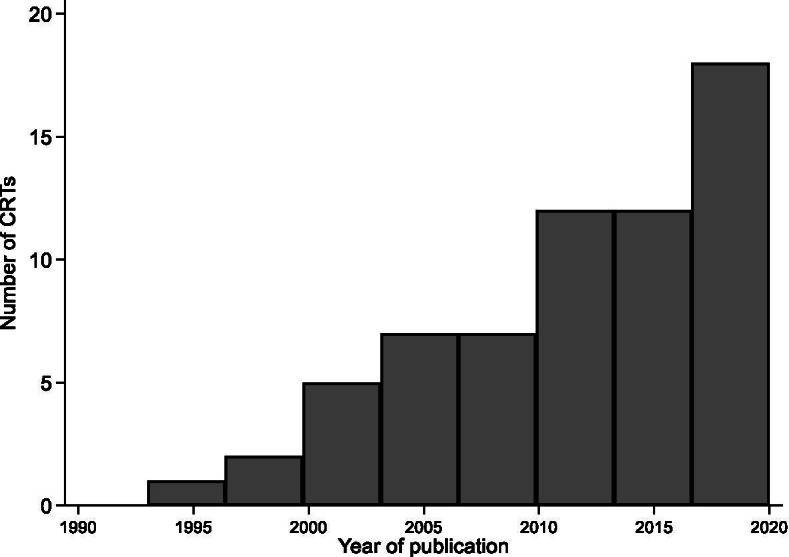
Published cluster randomised trials indexed in MEDLINE from inception to 30^th^ June 2020 (N = 64)

The included papers were published in 36 different journals, including: *British Medical Journal* (n = 9 papers); *BMC Public Health* (n = 4); *International Journal of Behavioural Nutrition and Physical Activity* (n = 4); *Archives of Disease in Childhood* (n = 3); *BMJ Open* (n = 3); *Journal of Epidemiology and Community Health* (n = 3); *Public Health Nutrition* (n = 3); and *The Lancet* (n = 3). The CRT design has been increasingly used in the UK school setting to evaluate health interventions for pupils since the first paper was published in 1993 (Fig. 2). Twenty three papers were published between 2001 and 2010, compared to 37 between January 2011 and June 2020.

Table 2 summarises the characteristics of included studies.

**Table 2:** Characteristics of included studies (N = 64)

Characteristic	N	Statistics
***Setting***		
Country	64	
England, n (%)		47 (73)
Scotland, n (%)		5 (8)
Wales, n (%)		3 (5)
Northern Ireland, n (%)		3 (5)
More than one country^a^, n (%)		6 (9)
Number of regions from which schools were drawn^b^	64	
One		40 (62)
Two		10 (16)
Three		1 (2)
Four		1 (2)
Unclear		12 (19)
School level	64	
Preschool only, n (%)		2 (3)
Primary only, n (%)		36 (56)
Secondary only, n (%)		24 (38)
Primary and Secondary, n (%)		2 (3)
School types that were included [83]^c^	44	
State, n (%)		41 (93)
Independent, n (%)		6 (14)
Academies, n (%)		2 (5)
Grammar, n (%)		2 (5)
Special, n (%)		2 (5)
Voluntary aided, n (%)		2 (5)
Foundation, n (%)		1 (2)
Faith, n (%)		1 (2)
***Intervention***		
Health area of intervention^d^	64	
Nutrition, n (%)		18 (28)
Physical activity, n (%)		15 (23)
Socioemotional function and its influences^e^, n (%)		15 (23)
Dental health, n (%)		7 (11)
Smoking, n (%)		5 (8)
Injury, n (%)		5 (8)
Sexual health, n (%)		3 (5)
Alcohol misuse, n (%)		2 (3)
Cancer, n (%)		1 (2)
Communication skills (for children with autism), n (%)		1 (2)
Health attitudes (breast feeding), n (%)		1 (2)
Level of prevention	64	
Primary prevention, n (%)		60 (94)
Secondary prevention, n (%)		4 (6)
Type of intervention [1]^f^	64	
Individual-cluster, n (%)		11 (17)
Professional-cluster, n (%)		33 (52)
External-cluster, n (%)		32 (50)
Cluster–cluster, n (%)		53 (83)
Multifaceted, n (%)		53 (83)
***Primary outcome***		
Primary outcome health area	64	
Socioemotional function and its influences^g^, n (%)		15 (23)
Nutrition, n (%)		10 (16)
Dental health, n (%)		7 (11)
Physical activity, n (%)		7 (11)
Obesity, n (%)		7 (11)
Smoking, n (%)		5 (8)
Injury, n (%)		3 (5)
Sexual health, n (%)		2 (3)
Obstetrics, n (%)		2 (3)
Alcohol misuse, n (%)		2 (3)
Cancer, n (%)		1 (2)
Communication skills (for children with autism), n (%)		1 (2)
Gross motor skills, n (%)		1 (2)
Safety, n (%)		1 (2)
Main reporter of primary outcome	64	
Pupil, n (%)		34 (53)
Researcher, n (%)		12 (19)
Dentist, n (%)		6 (9)
Teacher, n (%)		5 (8)
Parent, n (%)		4 (6)
Routine data, n (%)		2 (3)
Researcher and parent, n (%)		1 (2)
Primary outcome reporter blind to allocation status	64	
Yes, n (%)		18 (28)
No, n (%)		46 (72)
Primary outcome measurement was objective	64	
Yes, n (%)		14 (22)
No, n (%)		50 (78)
***Study design and analysis methods***		
Justification provided for randomising clusters	64	
Yes, n (%)		17 (27)
No, n (%)		47 (73)
Reason for randomising clusters	17	
To avoid contamination, n (%)		9 (53)
Intervention was delivered at the cluster level, n (%)		4 (24)
To avoid contamination and for logistical reasons, n (%)		2 (12)
To avoid contamination and avoid “selection bias”, n (%)		1 (6)
To avoid contamination and because intervention was delivered at the cluster level, n (%)		1 (6)
Unit of randomisation	64	
Schools, n (%)		56 (88)
Classes, n (%)		6 (9)
Year groups, n (%)		2 (3)
Number of trial arms	64	
Two, n (%)		55 (86)
Three, n (%)		5 (8)
Four, n (%)		4 (6)
Study design	64	
Parallel group, n (%)		61 (95)
Factorial, n (%)		3 (5)
Method used to sample schools	64	
All potentially eligible schools invited, n (%)		33 (52)
Random sample, n (%)		5 (8)
Purposive sample, n (%)		4 (6)
Convenience sample, n (%)		3 (5)
Mixed random/convenience sample, n (%)		1 (2)
Unclear, n (%)		18 (28)
Type of randomisation	64	
Completely randomised, n (%)		13 (20)
Stratified, n (%)		29 (45)
Matched, n (%)		8 (13)
Minimisation, n (%)		8 (13)
Constrained [84, 85], n (%)		6 (9)
Type of follow-up	64	
Cohort, n (%)		62 (97)
Repeated cross-sectional, n (%)		1 (2)
Mixed, n (%)		1 (2)
Number of follow-ups	64	
1, n (%)		32 (50)
2, n (%)		21 (33)
3, n (%)		6 (9)
4, n (%)		5 (8)
Length of follow-up	64	
Up to 6 months, n (%)		22 (34)
7 to 12 months, n (%)		19 (30)
13 to 18 months, n (%)		6 (9)
19 to 24 months, n (%)		8 (13)
25 to 36 months, n (%)		7 (11)
More than 36 months, n (%)		2 (3)
Participants recruited before clusters were randomised	64	
Yes, n (%)		21 (33)
No, n (%)		17 (27)
Unclear, n (%)		26 (41)
Baseline data collected before clusters were randomised	64	
Yes, n (%)		16 (25)
No, n (%)		27 (42)
Unclear, n (%)		21 (33)
Method of analysis	64	
Individual-level analysis that allows for clustering, n (%)		46 (72)
Cluster-level analysis, n (%)		10 (16)
Did not allow for clustering, n (%)		8 (12)
***Sample size calculation***		
Assumed school-level intra-cluster correlation coefficient of outcome, median (IQR; range)	37	0.05 (0.02 to 0.1; 0.005 to 0.175)
Assumed design effect, median (IQR; range)	36	2.21 (1.98 to 3.53; 1.22 to 8.11)
Study allowed for drop-out at cluster level	64	
Yes, n (%)		4 (6)
Not stated, n (%)		60 (94)
Study allowed for drop-out at individual level^h^	62	
Yes, n (%)		18 (29)
Not stated, n (%)		44 (71)
Target number of clusters, median (IQR; range)	46	30 (20 to 40; 4 to 160)
Target number of schools, median (IQR; range)	41	30 (20 to 42; 4 to 160)
Target number of individuals, median (IQR; range)^i^	45	964 (498 to 2000; 90 to 9000)
***Ethics and consent procedures***		
From whom was consent/assent sought for pupil participation?	64	
Parents and pupils, n (%)		40 (63)
Parents only, n (%)		15 (23)
Pupils only, n (%)		2 (3)
Not stated / Neither parent nor pupil, n (%)		7 (11)
Opt-out consent/assent procedure used for either parent/guardian or pupils	64	
Yes, n (%)		29 (45)
Not stated / No, n (%)		35 (55)
***Other study characteristics of methodological interest***		
Ethnicity: percentage of pupils that are White, median (IQR; range)	33	76.8 (51.5 to 86.2; 24 to 95.3)
Total number of clusters recruited, median (IQR; range)	62	31.5 (21 to 50; 4 to 486)
Total number of schools recruited, median (IQR; range)	63	29 (15 to 50; 4 to 486)
Total number of pupils recruited, median (IQR; range)^j^	60	1308 (604 to 3201; 17 to 27,435)
Percentage of clusters followed-up for primary outcome, median (IQR; range)	62	100 (92.5 to 100; 60.5 to 100)
Percentage of pupils followed-up for primary outcome, median (IQR; range)^k^	58	79.9 (64.1 to 87.5; 7.7 to 100)
Observed school-level intra-cluster correlation coefficient of primary outcome, median (IQR; range)	23	0.028 (0.017 to 0.12; 0.0005 to 0.21)

^a^Studies that included schools from more than one country in the United Kingdom

^b^English regions included: South West, South East (including Greater London), East of England, West Midlands, East Midlands, North West, North East, Yorkshire and The Humber, “Southern England”, “Central England” and “West of England”. Scottish regions included: Glasgow, Inverclyde, Tayside, Grampian, Lanarkshire, Lothian and Fife. Welsh regions included: North Wales, South West Wales and South East Wales. Northern Irish regions included: South Belfast, East Belfast, Ulster, Leinster, Connacht and Munster

^c^Some studies included more than one school type. This is the number of studies that included specific types of school. State schools receive funding through their local authority or directly from the government. The most common ones are local authority, foundation and voluntary aided school which are all funded by the local authority. Academies are run by government and not-for-profit trusts, and are independent of local authority. Grammar schools are run by local authorities but intake is based on assessment of the pupils’ academic ability. Special schools cater for pupils with special educational needs. Faith schools follow the national curriculum but can decide what they teach in religious studies. Independent schools follow the national curriculum but charge fees for attending pupils

^d^Some interventions targeted more than one health area

^e^Includes mental health, behaviour, ADHD, wellbeing, quality of life, bullying, social and emotional learning, and self-esteem

^f^Intervention type was summarised based on the typology described by Eldridge and colleagues [1]. ‘Individual-cluster’ interventions include components that are directed at individual participants (e.g. pupils) on whom outcomes are measured. ‘Professional-cluster’ interventions include components for training professionals in the cluster (e.g. teachers in schools) to deliver the intervention. ‘External-cluster’ interventions involve additional staff outside the cluster to deliver the intervention (e.g. researchers, trained facilitators). ‘Cluster–cluster’ interventions include components that necessarily have to be administered to entire clusters (e.g., school policy). ‘Multifaceted’ interventions include components across more than one of the ‘individual-cluster’, ‘professional-cluster’, ‘external-cluster’ and ‘cluster–cluster’ categories

^g^Includes mental health, behaviour, hyperactivity/inattention (ADHD), wellbeing, quality of life, bullying, social and emotional learning, and self-esteem (body image)

^h^Summary excludes the two CRTs that did not use the cohort design

^i^Summary excludes the two CRTs that did not use the cohort design

^j^Summary excludes the two CRTs that did not use the cohort design

^k^Summary excludes the two CRTs that did not use the cohort design

### Setting

Almost three quarters of the studies were conducted exclusively in England (n = 47; 73%); most studies (50 of the 52 studies that provided the data) took place in one or two geographic regions (e.g., West Midlands). Just over half the studies (56%) were based exclusively in primary schools (age 5–11 years), and 38% were exclusively in secondary schools (age 11–16 years). Of the 44 studies that reported information on the types [83] of schools recruited, 93% included state-funded schools.

### Intervention type

Eighteen (28%) studies evaluated interventions that targeted nutrition, 15 (23%) physical activity, 15 (23%) socioemotional function and its influences, 7 (11%) dental health, 5 (8%) smoking and 5 (8%) injury, amongst others. Physical health interventions are increasingly prominent (13 published since 2011 in contrast to just 2 prior to then). Of the 15 studies targeting socioemotional function and its influences, 13 were published since 2011, highlighting increasing use of the CRT design in this area. Of the 7 CRTs related to dental health, the most recent one was published in 2011. The vast majority of interventions were in primary prevention (94%).

In 53 (83%) studies, the intervention had at least one component that necessarily had to be administered to entire clusters (“cluster–cluster” interventions [1]). Such components often included educational lessons (e.g., classroom-based lessons [23], physical activity [43] and gardening [25]). Other less common components included breakfast clubs [46, 73], funding/resources [37], change in school policy [50] and advertisements [40]. Eleven (17%) studies had intervention components that directly targeted individual pupils (“individual-cluster” interventions [1]), such as the use of fluoride varnish [72]. Thirty three (52%) studies had “professional-cluster” interventions [1]: in 30 (47%) studies the teacher was either trained in or provided with guidance to deliver components of the intervention, in 3 studies pupils were trained to deliver peer-led intervention components [21, 26, 42], and in 1 study the school nurse was trained [66]. Half the studies (n = 32) had “external-cluster” interventions [1] where people external to the school delivered intervention components (e.g., researchers [23], trained facilitators [53], dental professionals [51], dance instructors [41] and student volunteers [47]).

Two studies [53, 78] had 2 control groups (one “usual care” and one active) and 16 (25%) used a delayed intervention (waitlist) design.

### Primary outcome

Health areas assessed by the primary outcomes are summarised in Table 2. In 53% of the studies pupils reported the primary outcome, with researchers reporting primary outcomes in 20%, teachers in 8%, and parents in 8%. In 28% of the studies the primary outcome reporter was blind to allocation status (some authors specifically commented on the challenges of blinding trial arm status [33, 36, 56, 60]), and 22% measured the outcome using an objective method.

### Study design and analysis methods

Explicit justification for use of the CRT design was only provided in 17 (27%) studies; the most common reason was to avoid contamination (13 studies altogether). Most studies (n = 56; 88%) randomised school clusters, while classes and year groups were allocated in 6 (9%) and 2 (3%) studies, respectively. Two authors said that in order to maintain power, classes were randomised instead of schools and that this may have led to contamination between the intervention and control arms [22, 28]. Nearly all studies used a parallel group design (n = 61; 95%); the remaining 3 used a factorial design [21, 37, 39]. Of the 46 studies with sufficient information to establish the approach used to sample schools, 33 initially invited all potentially eligible schools to participate, 5 used random sampling, 4 used purposive sampling, 3 used convenience sampling, and 1 used a mixed random/convenience sampling approach.

Eighty percent of studies reported using a restricted allocation method to balance cluster-level characteristics between the trial arms. Most commonly a measure of socio-economic status (SES) was balanced on (48%), with a third of studies (21/64) specifically balancing the allocation on the percentage of pupils eligible for free school meals. Other commonly-used balancing factors are described in Table 3. Few studies gave justification for their choice of balancing factors.

**Table 3:** Cluster-level characteristics used to balance the randomisation (N = 64)

Characteristic	Statistic
Deprivation (school or area in which school is based)	
Yes – Percentage of pupils eligible for free school meals, n (%)	21 (33)
Yes – Townsend Index [86]^a^, n (%)	2 (3)
Yes – Income Deprivation Affecting Children Index (IDACI) [87]^b^, n (%)	1 (2)
Yes – Index of Multiple Deprivation (IMD) [87]^c^, n (%)	1 (2)
Yes – Unspecified^d^, n (%)	6 (9)
Cluster size	
Yes, n (%)	23 (36)
Geographic area of school	
Yes, n (%)	13 (20)
Pupil ethnicity summary	
Yes, n (%)	5 (8)
Co-educational status of school Yes, n (%)	5 (8)
School performance	
Yes, n (%)	5 (8)
School type	
Yes, n (%)	2 (3)
Other^e^	
Yes, n (%)	24 (38)

^a^Townsend Index quantifies material deprivation within a population

^b^Income Deprivation Affecting Children Index (IDACI) is the proportion of all children aged 0 to 15 living in income deprived families in different local areas across England

^c^Index of Multiple Deprivation (IMD) measures relative deprivation for small areas (or neighbourhoods) in England

^d^Did not state which measure of deprivation used

^e^Other balancing factors include: Percentage of students who actively commuted to school; School; English-speaking versus Welsh-speaking school; Local sexual health services; Number of students in year group; Date of entry of school into study; School in urban versus rural area; Percentage of children speaking English as an additional language; Quality and quantity of current school sex education; Local authority; Percentage of pupils staying on after age 16 years; Special educational need status; Whether school has existing policy similar to the intervention; School expressed preference for allocation (control versus intervention versus no preference); Health-promoting school status; Percentage of children in year group of interest with no dental decay; Frequency and timetabling of personal, social, and health education lessons; Preferred timetabling of the intervention; Facilitator of the intervention (Regional Project Manager)

One of the challenges of CRTs is to avoid recruitment bias that might occur if participants are recruited after the clusters are randomised [88, 89]. One third (33%) of studies avoided this by recruiting pupils before the clusters were randomised; furthermore, 25% collected baseline data before randomisation. This information, however, was unclear in many studies (41% and 33%, respectively). Generally, insufficient information was provided on whether recruitment bias was avoided in studies where pupils were recruited after randomisation of clusters. A notable exception was one study [57] where recruitment bias was avoided because allocation was not revealed to the schools until after recruitment and baseline assessment.

Nearly all studies used the cohort design as their method of follow-up (n = 62, 97%), where the same pupils provided data at each study wave. One study used a repeated cross-sectional design where different pupils provided data at each wave [46], and one used an a priori mixed design incorporating elements of the cohort and repeated cross-sectional designs, with only a subset of participating pupils providing data at each wave [49].

Seventy two percent of studies analysed their data using individual-level methods that allow for clustering, 16% used cluster-level analysis methods, and 12% did not allow for clustering in their analysis.

### Sample size calculation

Seventy eight percent of studies accounted for clustering in their sample size calculation and 72% reported the ICC or coefficient of variation [90] that was assumed for the outcome. None of the studies made a degrees of freedom correction to the sample size calculation. Only two studies [57, 63] allowed for unequal cluster sizes in their sample size calculation, and only one of these [57] specified the anticipated variation in the number of pupils across clusters. The median (range) assumed ICC for school clusters was 0.05 (0.005 to 0.175) based on the 37 studies that provided these data. Of the 3 studies that specified the coefficient of variation of the outcome, 2 assumed it to be 0.2 [42, 60] and 1 assumed it to be 0.25 [19]. The median (range) assumed design effect was 2.21 (1.22 to 8.11). The median targeted sample size was 30 and 964 clusters and pupils, respectively. Most studies (94%) did not state whether their sample size calculation allowed for loss to follow-up of clusters.

### Ethics and consent procedures

Information regarding consent procedures was not well reported and consent for the participation of the cluster was often implied rather than explicitly detailed. In 63% of studies it was stated that both parents/guardians and pupils provided consent or assent for study participation. Forty five percent of studies reported that opt-out consent [14] from either the parent/guardian and/or the pupil was used for participation.

### Other study characteristics of methodological interest

A median (IQR) of 31.5 (21 to 50) clusters, 29 (15 to 50) schools and 1308 (604 to 3201) pupils were recruited. The CRT studies that used a cohort design and reported both targeted and achieved recruitment figures at the cluster (n = 45) and pupil (n = 43) levels achieved those recruitment targets in 89% and 77% of studies, respectively. Some authors noted challenges with recruitment at the cluster [45, 47, 50] and pupil [24, 55] levels. Based on the 33 studies that provided data, the median (IQR) percentage of pupils categorised as “White” was 76.8% (51.5% to 86.2%). Thirty out of 62 (48%) studies that provided information reported that at least one cluster was lost to follow-up. Missing data resulting from entire school drop-out was highlighted as a problem in some reports (e.g., [42, 48, 54]). The median follow-up at the pupil level was 79.9%.

**Fig. 3:**
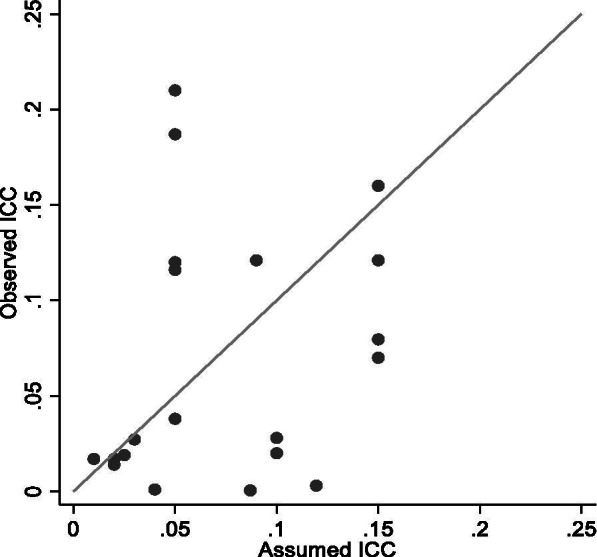
Observed ICC for primary outcomes versus ICC assumed in sample size calculation (N = 20)

Only 26 (41%) studies overall, and 18 of the 37 (49%) studies published after 2010, reported the ICC from the analysis of the primary outcome; the specific ICC values are reported in Table 4. The median (range) ICC for school clusters was 0.028 (0.0005 to 0.21). For many studies that reported both values there was a marked difference between the observed school-level ICC in the study data and the corresponding assumed value of the ICC in the sample size calculation (Fig. 3). The median (range) of the differences between the observed ICC and the assumed ICC was -0.006 (-0.117 to 0.16) indicating that: on average, the observed ICC was slightly smaller than the assumed ICC; at one extreme, the observed ICC in one study was 0.117 smaller than the assumed value [25]; and at the other extreme, the observed ICC in one study was 0.16 larger than the assumed value [68]. The intra-class correlation coefficient of agreement between the observed and assumed ICCs was 0.24.

**Table 4:** Reported intra-cluster correlation coefficients for primary outcomes (N = 26)

Author	Year	Cluster unit	Outcome	Health area	Outcome type	ICC estimate
Stallard [53]	2012	year group	Symptoms of low mood (depression)	socioemotional function	continuous	0.012
Chisholm [22]	2016	class	Stigma of mental illness	socioemotional function	continuous	0.1
Obsuth [71]	2017	school	School exclusion	socioemotional function	binary	0.028
Connolly [82]	2018	school	Prosocial behaviour	socioemotional function	continuous	0.116
Ford [32]	2019	school	Mental health / behaviour	socioemotional function	continuous	0.121
Axford [44]	2020	school	Victimisation (being bullied) occurring at least twice a month in the last 2 months	socioemotional function	binary	0.019
Campbell [26]	2008	school	Smoking in the past week	smoking	binary	0.017
Conner [74]	2019	school	Ever smoking	smoking	binary	0.017
McKay [24]	2018	school	Heavy episodic drinking in the previous 30 days (> = 6 units for males and > = 4.5 units for females)	alcohol misuse	binary	0.121
Croker [40]	2012	school	Child's eating habits	obesity	continuous	0.07
Fairclough [56]	2013	school	Waist circumference (cm)	obesity	continuous	0.06
Lloyd [57]	2018	school	BMI z score	obesity	continuous	0.014
Breheny [43]	2020	school	BMI z-score at 12 months	obesity	continuous	0.001
Jago [41]	2015	school	Mean weekday minutes of moderate to vigorous physical activity per day	physical activity	continuous	0.0005
Harrington [58]	2018	school	Minutes per day of moderate- to vigorous physical activity	physical activity	continuous	0.02
Norris [67]	2018	school	Sedentary behaviour during the school day in minutes	physical activity	continuous	0.080
James^a^ [28]	2004	class	Consumption of carbonated drinks over 3 days (in glasses)	nutrition	continuous	-0.009
Christian [25]	2014	school	Combined daily fruit and vegetable intake (grams per day)	nutrition	continuous	0.003
Redmond [81]	1999	school	Proportion of teeth sites with caries at 6 months	dental health	continuous	0.16
Worthington [31]	2001	school	Plaque score	dental health	continuous	0.023
Milsom [59]	2006	school	Whether the child has active caries in their first permanent molars	dental health	binary	0.027
Mulvaney [68]	2006	school	Use of visibility aid (reflective and fluorescent slap wrap) while cycling	injury	binary	0.21
Kendrick [79]	2007	school	Knowledge score for fire and burn prevention	safety	continuous	0.187
Hubbard [76]	2016	school	Number of recognised cancer warning signs	cancer	continuous	0.038
Henderson [27]	2007	school	Terminations of pregnancy by age 20	obstetrics	count	0.005
Giles [23]	2014	school	Intention to breastfeed	obstetrics	continuous	0.12

^a^The estimated intra-cluster correlation coefficient in James (2004) was negative. True negative values are generally considered implausible in the context of cluster randomised trials

Seven studies [24, 26, 44, 59, 68, 71, 74] that reported ICCs had a binary primary outcome, but none of these stated whether the ICC was calculated on the proportions scale or the logistic scale [3]. It is possible that five of these studies [24, 26, 68, 71, 74] that used mixed effects (“multi-level”) models [91] to analyse the data reported the ICC on the logistic scale, which could potentially account for some of the differences between the observed and assumed ICCs. Further scrutiny of the data, however, revealed marked differences for only two of the aforementioned studies: 0.21 for the observed ICC versus 0.05 for the assumed ICC in Mulvaney and colleagues [68], and 0.028 versus 0.1, respectively, in Obsuth and colleagues [71].

## Discussion

The number of UK school-based CRTs evaluating the effects of interventions on pupil health outcomes has increased in recent years, reflecting growing recognition of the role that schools can play in improving the health of children [10, 92–95]. The findings of this systematic review indicate a number of methodological considerations that are worthy of reflection.

### Interpretation

Seventy two percent of the studies reported the level of clustering assumed in their sample size calculation, a little more than the 62% observed in a 2015 review of the reporting of sample size calculations in CRTs [96]. Our review found that the observed ICC in the study data often differed markedly from the ICC assumed in the sample size calculation. This will be partly due to sampling variation and adjustment for prognostic factors in the analysis, but it may also reflect the lack of availability of good estimates of the ICC at the time of sample size calculation. Knowledge of the ICC for pupil health outcomes in the school setting is less well established than for patient health outcomes in the primary care setting where general practices are allocated as clusters [1, 97]. It has been reported that general practice-level ICCs for health outcomes are generally less than 0.05 [98]; in our review, only 13 of 23 studies that randomised school clusters and reported observed ICCs had values that were less than 0.05. School-based ICC estimates are widely available for educational outcomes [99], but these are markedly higher than those reported in this review for pupil health outcomes; this is to be expected given that the primary role of the school is to provide education. The importance of reporting ICCs from study data for planning future similar CRTs has long been established [100] and the 2012 CONSORT extension to CRTs includes a specific reporting item for this [101]. Only two-fifths (41%) of studies in this review, however, reported the ICC for the primary outcome; this figure rises to 48% (16/33) for studies published after 2012. Improved reporting of the ICC in the increasing number of CRTs in the school-based setting, and further papers written specifically to report ICCs [102, 103], will provide valuable knowledge. This review focussed on CRTs in the UK setting; a useful area to investigate is the extent to which school-based ICC estimates for health outcomes from other countries (e.g., [102, 104]) are similar to those in the UK.

Representativeness of school and pupil characteristics in school-based trials is important for external validity and inclusiveness. For most studies in this review, schools were recruited from only one or two geographic regions/counties. A median 23% of participating pupils were in a minority ethnic group, lower than the national percentages reported by the UK Department for Education (33.5% of primary school pupils and 31.3% of secondary school pupils) [105]. The study reports generally provided little information on specific aspects of the recruitment process, such as why some schools declined to participate and details of their characteristics. Many of the studies evaluated interventions that involved classroom lessons and necessitated teachers being trained to deliver the intervention. Additionally, the teachers reported pupil outcomes in some studies [32, 34, 60, 73, 82]. Insufficient school resources to deliver the intervention and the wider trial may be a barrier to participation and result in lack of representation of certain types of schools.

Eighty percent of the studies used some form of restricted allocation to balance the randomisation on cluster-level characteristics, which is higher than previous methodological reviews of CRTs [106–109]. The percentage of pupils in the school that are eligible for free school meals was often used as a balancing factor, perhaps partly because this information is readily available from the UK Department for Education [110]. School characteristics that are predictive of the study outcomes, account for within-cluster correlation or influence effectiveness of the intervention are candidates on which to balance the randomisation [1, 111]; previous school-based CRTs could be used to identify such factors.

### Strengths

This systematic review used a defined search strategy tailored to identify school-based CRTs. The strategy was developed following an iterative process and allowed us to achieve the right balance of sensitivity and specificity relevant to our available resources. Identifying reports of CRTs is a challenge given that many articles do not used the term ‘cluster’ in their title or abstract. Therefore, a search strategy was used which included terms such as ‘group’ and ‘community’ to improve sensitivity. The ‘School’ MeSH term was also used to identify publications that randomised any type of school-related unit. The piloting of our screening procedure and data extraction were conducted by two independent reviewers, improving accuracy. The review identified school-based CRTs with interventions spanning a variety of different health conditions/areas.

### Limitations

A potential limitation of the review is that the search was limited to one database. MEDLINE was used because the focus of the review was on describing the characteristics of trials that evaluate the impact of health interventions on pupil’s health outcomes, but it is possible that we have not identified eligible publications that are not indexed in MEDLINE. Translating our search in the EMBASE, DARE, PsycINFO and ERIC databases for potential includes published in the last 3 years, however, revealed only one additional eligible school-based CRT.

Given resource constraints, we focussed the review on the UK, making the decision to collect rich data on CRT methodology in a single education system. As a result, the findings are readily applicable to a specific context. Despite being focussed on the UK, the findings of this review will be of global interest. Other high income countries, such as Australia, have a similar school system to the UK, and many of our findings may be applicable in those settings. Furthermore, some of the methodological challenges in the design of CRTs will be similar across different settings.

### Future directions

The results provide a summary of the methodological characteristics of school-based CRTs with pupil health outcomes in the UK. To our knowledge, there has been no systematic review of the characteristics of school-based CRTs for evaluating interventions for improving education outcomes, despite the fact that the use of the CRT design is more established in that area. A comparison of methodology between health-based CRTs and education-based CRTs in the school setting would be valuable to both areas. The results in our review indicate that better information on the ICC is needed to design school-based CRTs with health outcomes. Cataloguing of ICCs from previous studies will help researchers choose better values for the assumed ICC when calculating sample size.

## Conclusions

CRTs are increasingly used in the school setting for evaluating interventions for improving children’s health and wellbeing. The emerging pool of published trials in the UK provides investigators and methodologists with relevant experiential knowledge for the design of future similar studies. This review of school-based CRTs has highlighted the need for more information on the ICCs to calculate the required sample size. Better reporting of the recruitment process in CRTs will help to identify common barriers to obtaining representative samples of schools and pupils. Finally, previous school-based CRTs may provide a useful source of data to identify the school-level characteristics that are strong predictors of pupil health outcomes and, therefore, potentially good factors on which to balance the randomisation.

## Data Availability

The datasets generated and/or analysed during the current study are not publicly available because they are also being used for a wider ongoing programme of research but are available from the corresponding author on reasonable request.

## Abbreviations

CRT: Cluster randomised trial
DARE: Database of Abstracts of Reviews of Effects
EMBASE: Excerpta Medica Database
ERIC: Education Resources Information Center
ICC: Intra-cluster correlation coefficient
IQR: Interquartile range
MeSH: Medical Subject Headings
PRISMA: Preferred Reporting Items for Systematic Reviews and Meta-Analyses
PsycINFO: Psychological Information Database
SES: Socio-economic status
UK: United Kingdom
